# Data extraction from free-text stroke CT reports using GPT-4o and Llama-3.3-70B: the impact of annotation guidelines

**DOI:** 10.1186/s41747-025-00600-2

**Published:** 2025-06-19

**Authors:** Jonas Wihl, Enrike Rosenkranz, Severin Schramm, Cornelius Berberich, Michael Griessmair, Piotr Woźnicki, Francisco Pinto, Sebastian Ziegelmayer, Lisa C. Adams, Keno K. Bressem, Jan S. Kirschke, Claus Zimmer, Benedikt Wiestler, Dennis Hedderich, Su Hwan Kim

**Affiliations:** 1 Department of Diagnostic and Interventional Neuroradiology, TUM University Hospital, School of Medicine and Health, Technical University of Munich, Munich, Germany; 2 Department of Diagnostic, Interventional and Pediatric Radiology, Inselspital Bern, University of Bern, Bern, Switzerland; 3 Smart Reporting GmbH, Munich, Germany; 4 Department of Diagnostic and Interventional Radiology, TUM University Hospital, School of Medicine and Health, Technical University of Munich, Munich, Germany; 5 Department of Cardiovascular Radiology and Nuclear Medicine, German Heart Center Munich, School of Medicine and Health, Technical University of Munich, Munich, Germany; 6 AI for Image-Guided Diagnosis and Therapy, School of Medicine and Health, Technical University of Munich, Munich, Germany

**Keywords:** Artificial intelligence, Information storage and retrieval, Large language models, Stroke, Tomography (x-ray computed)

## Abstract

**Background:**

To evaluate the impact of an annotation guideline on the performance of large language models (LLMs) in extracting data from stroke computed tomography (CT) reports.

**Methods:**

The performance of GPT-4o and Llama-3.3-70B in extracting ten imaging findings from stroke CT reports was assessed in two datasets from a single academic stroke center. Dataset A (*n* = 200) was a stratified cohort including various pathological findings, whereas dataset B (*n* = 100) was a consecutive cohort. Initially, an annotation guideline providing clear data extraction instructions was designed based on a review of cases with inter-annotator disagreements in dataset A. For each LLM, data extraction was performed under two conditions: with the annotation guideline included in the prompt and without it.

**Results:**

GPT-4o consistently demonstrated superior performance over Llama-3.3-70B under identical conditions, with micro-averaged precision ranging from 0.83 to 0.95 for GPT-4o and from 0.65 to 0.86 for Llama-3.3-70B. Across both models and both datasets, incorporating the annotation guideline into the LLM input resulted in higher precision rates, while recall rates largely remained stable. In dataset B, the precision of GPT-4o and Llama-3-70B improved from 0.83 to 0.95 and from 0.87 to 0.94, respectively. Overall classification performance with and without the annotation guideline was significantly different in five out of six conditions.

**Conclusion:**

GPT-4o and Llama-3.3-70B show promising performance in extracting imaging findings from stroke CT reports, although GPT-4o steadily outperformed Llama-3.3-70B. We also provide evidence that well-defined annotation guidelines can enhance LLM data extraction accuracy.

**Relevance statement:**

Annotation guidelines can improve the accuracy of LLMs in extracting findings from radiological reports, potentially optimizing data extraction for specific downstream applications.

**Key Points:**

- LLMs have utility in data extraction from radiology reports, but the role of annotation guidelines remains underexplored.
- Data extraction accuracy from stroke CT reports by GPT-4o and Llama-3.3-70B improved when well-defined annotation guidelines were incorporated into the model prompt.
- Well-defined annotation guidelines can improve the accuracy of LLMs in extracting imaging findings from radiological reports.

**Graphical Abstract:**

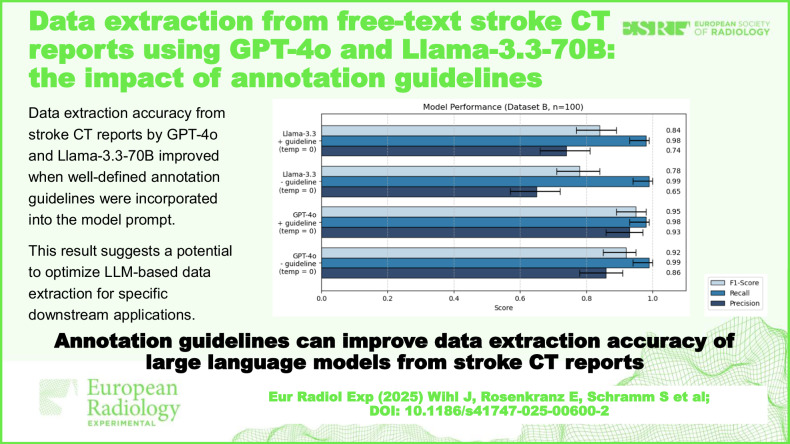

## Background

Computed tomography (CT) imaging in suspected acute stroke, including unenhanced CT (NECT), CT angiography, and CT perfusion, provides critical insights into the type of stroke (ischemic or hemorrhagic), the presence of vessel occlusions, and the extent of brain damage. The findings recorded in radiological reports are pivotal in determining eligibility for intravenous thrombolysis or mechanical thrombectomy [1]. Importantly, the data contained in these reports holds enormous value beyond its utility in clinical decision-making, enabling studies on epidemiology [2], pathophysiology [3], treatment efficacy [4, 5], and patient outcomes [6, 7]. Key variables can further be utilized as labels for training machine learning algorithms for tasks such as detecting large vessel occlusion [8] or automatic evaluation of the Alberta Stroke Program Early CT Score (ASPECTS) [9]. Imaging findings also play a crucial role in national stroke registries aiming to monitor and improve the quality of stroke care [10, 11].

Yet, given that most radiology reports still consist of prose and lack standardized terminology, analysis of reported findings previously necessitated labor-intensive manual annotations by experts, limiting scalability [12]. Natural language processing systems based on machine learning have shown promising results in automating information extraction from radiology reports, but were limited by the scarcity of annotated training data and the variability of reports [13–15].

Recently, large language models (LLMs) have demonstrated great potential in overcoming these limitations. In radiology, LLMs have shown significant promise in tasks such as report generation [16, 17], report translation [18], and differential diagnosis (DDx) [19, 20]. The data extraction performance of LLMs has been evaluated in different modalities, ranging from x-ray to interventional angiography. Notably, both proprietary [21–23] and open-source LLMs [24, 25] have been assessed, with open-source models offering the advantage of local data processing, enhancing patient data privacy. In models of both categories, accuracy levels of more than 90% of correctly extracted parameters have been reported [22, 24], demonstrating their potential utility.

However, methodological inconsistencies in these types of studies pose challenges. For instance, many studies relied on manual annotations by only a single annotator [21, 22, 26], making the reference standard prone to subjective bias and human error. In addition, a scoping review by Reichenpfader et al [27] pointed out that only 9% (3/34) of studies reported annotation guidelines, unveiling a frequent lack of standardization and transparency in the annotation process. Moreover, many studies modeled findings in radiology reports as simple binary variables, which fail to capture the nuanced levels of diagnostic uncertainty expressed in the textual descriptions [24, 27, 28].

The aim of this study was to evaluate the performance of LLMs in data extraction from stroke CT reports, with or without annotation guidelines supplied as additional input.

## Methods

This retrospective study was approved by the Institutional Review Board of the Technical University of Munich (TUM), and the need for informed consent was waived.

### Datasets

This study employed two datasets from a single German academic institution with a comprehensive stroke center. Both included patients who underwent a CT examination in suspected acute stroke, featuring either unenhanced CT with CT angiography only, or an additional CT perfusion. Reports were available in the German language.

Dataset A (*n* = 200) was a stratified cohort comprising five purposively sampled subgroups with the following imaging findings each (exam dates ranging from June 14, 2022 to July 14, 2024): ischemic stroke of the anterior circulation (*n* = 40), ischemic stroke of the posterior circulation (*n* = 40), extracranial pathology (*e.g*., carotid stenosis) (*n* = 40), intracranial hemorrhage (*n* = 40), and miscellaneous pathologies (*n* = 40). Covering various pathologies, this dataset served as the basis for creating a comprehensive annotation guideline. Dataset B (*n* = 100) contained a chronologically collected, consecutive cohort between August 1, 2024, and September 14, 2024.

Prior to conducting the LLM queries, reports from both datasets were manually reviewed and curated by S.H.K. and J.W. to remove potentially identifying information (*e.g.*, names, examination dates).

A formal sample size calculation was not performed, given the exploratory nature of the evaluation and the absence of prior literature providing comparable effect sizes.

### Data extraction parameters

**Fig. 1:**
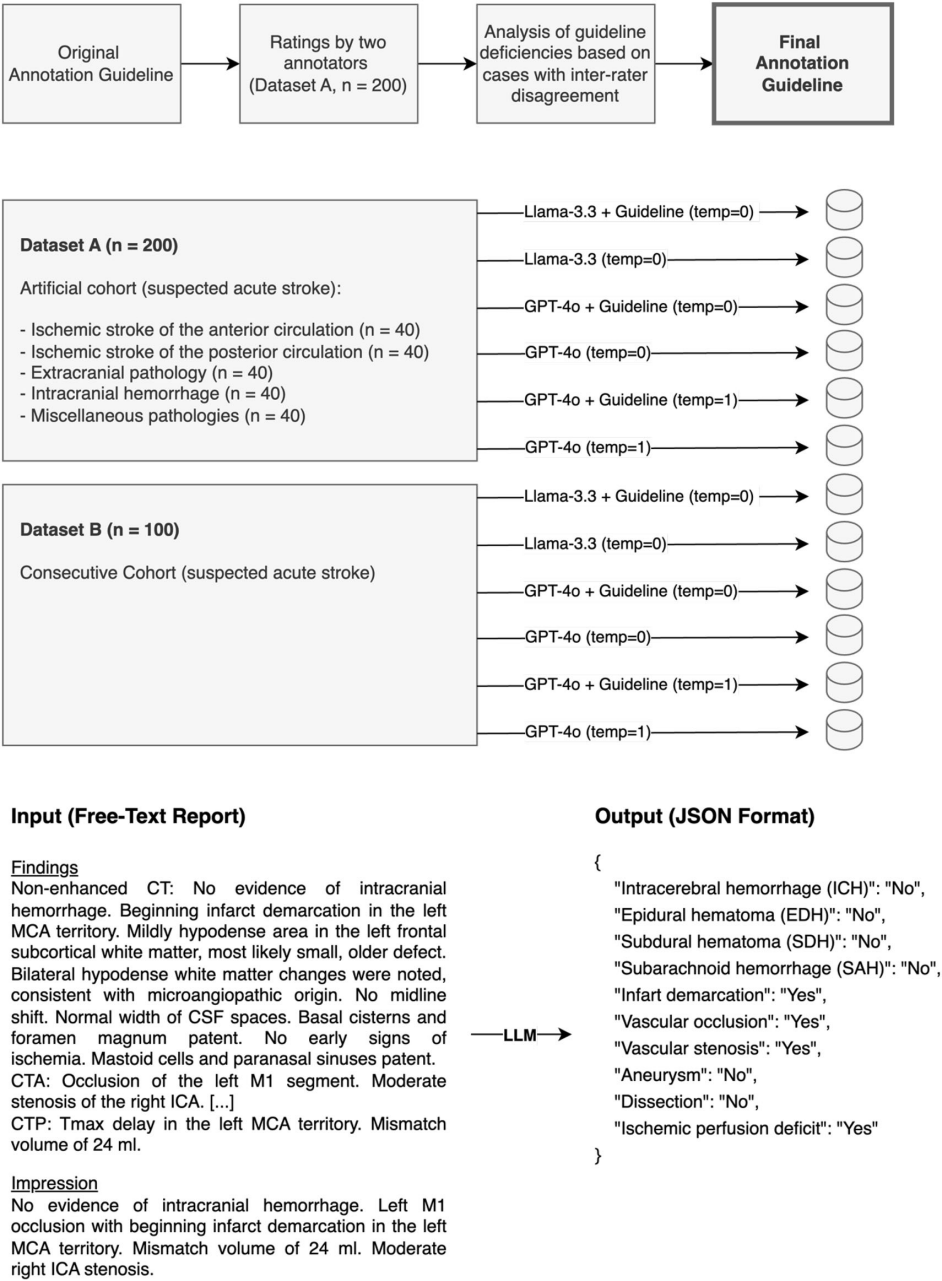
Study design. Initially, two raters annotated dataset A using a preliminary annotation guideline with a few general instructions. Based on the guideline deficiencies uncovered based on a review of cases with inter-rater disagreement, an addendum was appended to the original document, forming the final annotation guideline. The data extraction performance of GPT-4o and Llama-3.3-70B with and without the annotation guideline was evaluated in dataset A and additionally in another dataset (dataset B) that was not used to formulate the annotation guideline. At the bottom, a fictional CT report in English is shown along with the data parameters extracted from it in JSON format to illustrate the methodology

A template with the following ten imaging findings was created and represented in JavaScript Object Notation (JSON) format (Fig. 1): intracerebral hemorrhage (ICH), epidural hemorrhage, subdural hemorrhage, subarachnoid hemorrhage (SAH), infarct demarcation, vascular occlusion, vascular stenosis, aneurysm, dissection, and perfusion deficit.

### Manual annotations and annotation guidelines

A prototypical user interface (provided by Smart Reporting GmbH, Munich, Germany) was used to perform manual data entries. One radiology resident with two years of dedicated neuroradiology experience (S.H.K.) and one fourth-year medical student (J.W.) independently annotated dataset A (*n* = 200). A brief annotation guideline with general instructions (*e.g*., handling of missing data) defined by SHK was followed by both annotators. During the annotation process of dataset A, ambiguous instances were identified and recorded by the raters.

S.H.K. and D.H. (board-certified neuroradiologists with 10 years of experience) reviewed cases with inter-rater disagreement in dataset A, and added an addendum to the original annotation guideline addressing the identified edge cases. The resulting annotation guideline included example phrases and their correct labels. Such approaches, where LLMs are provided few examples of a task to guide the model’s output, are also known as ‘few-shot prompting’ [29]. Manual annotations of dataset A were revised according to the final guideline. Manual annotations of dataset B were conducted by S.H.K. and J.W. according to the same final guideline, and cases of inter-rater disagreement were resolved by D.H. Findings not mentioned in the report were considered absent. This reasoning principle, whereby anything not explicitly stated is assumed to be false or absent, is also known as the “closed-world assumption” [30]. Findings with uncertainty descriptors (“possible”, “DDx”, etc.) indicating no clear positive or negative tendency were labeled as “unknown” by annotators and omitted from the LLM data extraction analysis.

### LLM infrastructure

Generative pre-trained transformer 4 omni (GPT-4o) (‘gpt-4o-2024-08-06’) by OpenAI [31] and Large Language Model Meta AI (Llama)—Llama-3.3-70B (‘Llama-3.3-70B-Instruct’) by Meta [32] were chosen as representative state-of-the-art proprietary and open-source LLMs, each at the time of the study. GPT-4o was accessed via OpenAI’s application programming interface (API) (https://platform.openai.com/docs/models#gpt-4o). Llama-3.3-70B was deployed in a local environment utilizing the Python library “llama-cpp-python”, which provides compressed, less memory-intensive LLM instances (‘quantization’). A quantization factor of Q4_K_M was chosen to allow full GPU offloading. A single NVIDIA Quadro RTX 8000 with 48 GB of video memory was used for local inferences.

For both GPT-4o and Llama-3.3-70B, the model temperature was set to 0.0 to ensure deterministic results. To explore the impact of temperature settings on data extraction performance, GPT-4o queries were additionally run with the default temperature setting of 1. Our scripts for executing both models are publicly available in our repository at: https://github.com/shk03/stroke_llm_data_extraction.

### LLM queries

For both models, queries were performed with and without annotation guidelines. The base prompt was defined as follows (translated from German to English):

“*Extract the information provided in the radiological report in the format of a JSON file*.

*Each of the ten parameters should be evaluated as ‘Yes’ or ‘No.’ Findings that are not mentioned are considered absent and should be evaluated as ‘No*.’

*Please take the following guidelines into account: // only included in the ‘with guideline’ group*

*{annotation_guidelines} // only included in the ‘with guideline’ group*

*The JSON file should have the following structure*:

*{json_schema}*

*Here is the report from which the information should be extracted*:

*{report_text}*”

Queries with a temperature of 0 were conducted only once, assuming that this setting would lead to almost fully deterministic results. In contrast, GPT-4o queries with a temperature of 1 were repeated three times each to account for probabilistic variations. Execution times for LLM queries were recorded.

### GPT-4o's performance in diagnostic certainty assessment

In an additional experiment, the ability of GPT-4o to correctly evaluate diagnostic uncertainty of report content was evaluated in dataset B, using the default temperature setting of 1. GPT-4o was instructed to classify the ten parameters into one of the following five categories: ‘certainly absent’, ‘unlikely’, ‘possible’, ‘likely’, ‘certainly present’. Accuracy was rated against manual annotations by a single annotator (J.W.).

### Analysis

Statistical analyses and data visualizations were performed using Python (Version 3.11.8). To calculate accuracy metrics for GPT-4o queries with a temperature of 1, the mode of labels across three repetitions was used. The probabilistic variation was quantified as the percentage of cases producing consistent results across repetitions. For all conditions, precision (= positive predictive value), recall (= sensitivity), F1 (= harmonic mean of precision and recall), specificity, and negative predictive value were reported. Aggregated metrics across all parameters were computed using microaveraging, which consolidates true positives, false positives, and false negatives globally. Confidence intervals for precision and recall were determined using the Wilson score method [33]. The resulting lower and upper bounds were used to approximate the confidence interval of the F1 score. Overall classification performances were compared between groups with and without the annotation guideline using the McNemar test. A *p*-value < 0.05 was considered statistically significant. Correction for multiple testing was not performed, given the exploratory nature of the study.

## Results

### Cohort overview

An overview of the patient cohorts is presented in Table 1. Patients in both datasets had a median age of 79 (dataset A: interquartile range 72–85; dataset B: IQR 65–84), and an equal sex distribution (50.0 and 51.0% females each). Due to its purposive selection, dataset A exhibited a higher proportion of pathological findings than the consecutive dataset B, with particularly high occurrences of vascular occlusions (A: 43.0%, B: 21.0%), stenoses (A: 38.5%, B: 26.0%) and perfusion deficits (A: 42.5%, B: 25.0%). Intracranial hemorrhages were relatively rare, with ICHs being most common (A: 8.5%, B: 4.0%). Similarly, aneurysms (A: 4.5%, B: 4.0%) and arterial dissections (A: 1.5%, B: 1.0%) were found only on rare occasions.

**Table 1:** Cohort overview

	Dataset A (*n* = 200)	Dataset B (*n* = 100)
Demographics		
Median age (interquartile range), years	79 (72–85)	79 (65–84)
Female sex—no. (%)	100 (50.0)	51 (51.0)
Findings [frequency (%)]		
ICH	17 (8.5)	4 (4.0)
Epidural hematoma (EDH)	1 (0.5)	0 (0.0)
Subdural hematoma (SDH)	16 (8.0)	2 (2.0)
SAH	16 (8.0)	1 (1.0)
Infarction demarcation	35 (17.5)	11 (11.0)
Occlusion	86 (43.0)	21 (21.0)
Stenosis	77 (38.5)	26 (26.0)
Aneurysm	9 (4.5)	4 (4.0)
Dissection	3 (1.5)	1 (1.0)
Ischemic perfusion deficit	85 (42.5)	25 (25.0)

Dataset A was a stratified cohort with 40 cases of the following five subgroups each: ischemic stroke of the anterior circulation, ischemic stroke of the posterior circulation, extracranial pathology, intracranial hemorrhage, and miscellaneous pathologies. Dataset B was a consecutive cohort of cases with suspected acute stroke

### Annotation guidelines

The annotation guideline is presented in Table 2. The original guideline was used by the raters during initial annotation of dataset A, and contained general instructions on handling descriptive findings, indicators of diagnostic uncertainty, and contradictions within the report.

**Table 2:** Annotation guideline

Version	Type	Instruction
Original	General	No interpretation of findings A finding is only deemed present if it is explicitly mentioned. Examples: - Hypodensity in the parenchyma is not equivalent to infarct demarcation. - “Calcifications” or “plaques” are not equivalent to stenosis. - “Intraluminal thrombus” is not equivalent to stenosis. - “Loss of corticomedullary differentiation” is not equivalent to infarct demarcation. - “Contrast agent discontinuation,” “vessel discontinuation,” or “missing vascular contrast” is equivalent to vessel occlusion. - If the ASPECTS score is below 10, it is classified as infarct demarcation.
Original	General	Diagnostic certainty - Findings with a positive tendency (*e.g.*, “most likely,” “suspicion of,” “probable”) are classified as “Yes”. - Findings with a negative tendency (*e.g.*, “unlikely,” “secondary consideration”) are classified as “No”.
Original	General	Contradictions - In case of contradictions between the “Findings” and “Impression” sections, the “Impression” is considered decisive, and the finding is classified accordingly.
Addendum	General	Exclusion of previous findings and medical history - Previous findings that are no longer present are not considered. - Previous medical history is not considered.
Addendum	General	Location of findings - A finding shall be considered present if at least one location is affected. For example, “stenosis” shall be evaluated as “Yes” if a stenosis is described in at least one location.
Addendum	Specific	Infarct demarcation - “Infarct demarcation” encompasses only acute changes. Chronic infarcts or post-ischemic changes shall not be counted.
Addendum	Specific	Aneurysms - “Aneurysm” refers exclusively to true intracranial aneurysms. Aortic aneurysms or pseudoaneurysms shall not be classified as aneurysms.
Addendum	Specific	Perfusion deficit - A Tmax delay shall be considered a perfusion deficit. - Perfusion deficits do not require a mismatch. - Only perfusion deficits attributed to ischemia in the report shall be counted. Perfusion deficits due to artifacts or other causes shall not be counted.
Addendum	Specific	Stenosis/occlusion - In cases where a high-grade stenosis or occlusion is indicated (“stenosis/occlusion”), “stenosis” shall be evaluated as present and “occlusion” as “No.” - Vascular irregularities shall not be classified as stenosis. - Vascular occlusion refers exclusively to arterial occlusions (excluding sinus or cerebral venous thrombosis). - Pre-existing occlusions shall also be classified as vascular occlusions.

Annotators agreed in 96.0% (1,920/2,000) of data points in dataset A (Cohen’s kappa κ = 0.852). A thorough review of cases with inter-annotator disagreement (4.0%; 80/2,000) revealed 1.3% (26/2,000) of discrepancies originating from unclear guideline instructions, as opposed to 2.7% (54/2,000) of cases resulting from careless labeling errors by the annotators. To address the guideline deficiencies identified from a review of data points with inter-rater agreement in dataset A, an addendum was created that contained both general instructions (*e.g*., handling previous findings) and directions on classifying specific edge cases (*e.g.*, not counting pseudoaneurysms as aneurysms). Using a few-shot prompting approach, potentially ambiguous instructions in the annotation guideline were complemented with one or more example expressions, along with the correct label. For instance, one instruction stated: “A finding is only deemed present if it is explicitly mentioned.” In addition, the following example was included to illustrate the instruction: “Hypodensity in the parenchyma is not equivalent to infarct demarcation.” The final annotation guideline containing the original instructions and addendum was provided to GPT-4o and Llama-3.3-70B.

### Model performance

We excluded 1.7% (34/2,000) and 1.4% (14/1,000) of data points from dataset A and dataset B, respectively, as the report text indicated diagnostic uncertainty without a clear positive or negative tendency (*e.g*., “possible”, “DDx”) (Supplements 1 and 2).

Overall, GPT-4o consistently demonstrated superior performance over Llama-3.3-70B under identical conditions, with microaveraged precision (= positive predictive value) ranging from 0.86 to 0.95 for GPT-4o and from 0.65 to 0.86 for Llama-3.3-70B. In dataset B, GPT-4o and Llama-3.3-70B (temperature = 0) yielded a precision of 0.95 and 0.74, each, in the presence of the annotation guideline, while both exhibited equal recall values (= sensitivity; both 0.98).

**Fig. 2:**
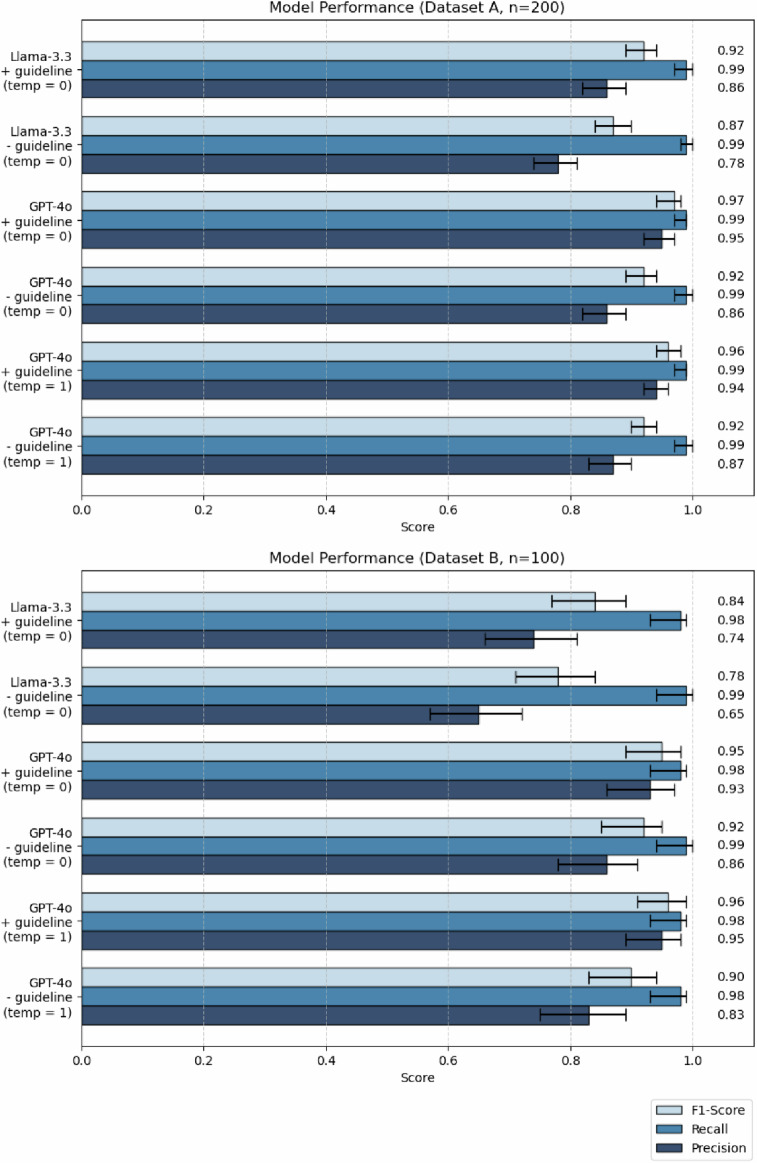
Data extraction performance of GPT-4o and Llama-3.3-70B across all parameters. Metrics for GPT-4o queries with a temperature setting of 1 were calculated based on the mode across three repetitions, whereas the remaining queries were run only once (with a temperature setting of 0.0). Error bars indicate 95% confidence intervals. The 1.7% (34/2,000) and 1.4% (14/1,000) of data points were excluded from dataset A and dataset B, each, as the report text indicated diagnostic uncertainty without a clear positive or negative tendency (expressions such as “possible”, “DDx”). Precision: positive predictive value. Recall: sensitivity. F1-Score: harmonic mean of precision and recall

Across all conditions, higher precision rates were observed when the annotation guideline was included in the prompt. The precision of GPT-4o (temperature = 1) improved from 0.87 to 0.94 in dataset A (*p* < 0.001) and from 0.83 to 0.95 in dataset B (*p* = 0.006). When using a temperature of 0, GPT-4o’s precision increases from 0.86 to 0.95 in dataset A (*p* < 0.001) and from 0.86 to 0.93 in dataset B, although the difference was not significant (*p* = 0.390). Similarly, the precision of Llama-3.3 (temperature = 0) rose from 0.78 to 0.86 (*p* < 0.001) in dataset A and from 0.87 to 0.94 in dataset B (*p* = 0.001). In contrast, recall rates largely remained stable, with values ranging from 0.98 to 0.99 in all conditions (Fig. 2).

Temperature settings of GPT-4o had only a minor impact on data extraction performance when the remaining conditions were equal. The largest difference in precision was seen in dataset B in the absence of guidelines, where an increase in the temperature resulted in a small drop in precision from 0.86 (temperature = 0) to 0.83 (temperature = 1).

Detailed variable-level metrics of GPT-4o in dataset B (temperature = 1) are presented in Table 3. Metrics varied widely, particularly in findings with low prevalence in the given dataset. The greatest increase in precision through the guideline adoption was seen in infarct demarcation (0.59–1.00), subdural hematoma (SDH) (0.67–1.00), and vascular stenosis (0.84–0.96). Exemplary cases where the annotation guideline influenced the LLM ratings are shown in Table 4. A review of data points where the LLM ratings were incorrect in both conditions showed that in some cases, the instruction to exclude previous findings (*e.g*., “status post”, “previously described […]”) was not followed. Granular variable-level metrics of remaining groups, along with specificity and negative predictive value, are provided in Supplements 3–13.

**Table 3:** Data extraction performance of GPT-4o (temperature = 1) with and without annotation guideline in dataset B (*n* = 100)

Variable	Dataset B: GPT-4o − Guideline (Temperature = 1)			Dataset B: GPT-4o + Guideline (Temperature = 1)		
	Precision	Recall	F1-score	Precision	Recall	F1-score
ICH	0.80 (0.38–0.96)	1.00 (0.51–1.00)	0.89 (0.43–0.98)	0.80 (0.38–0.96)	1.00 (0.51–1.00)	0.89 (0.43–0.98)
EDH	0.00 (0.00–0.00)	0.00 (0.00–0.00)	0.00 (0.00–0.00)	0.00 (0.00–0.00)	0.00 (0.00–0.00)	0.00 (0.00–0.00)
SDH	0.67 (0.21–0.94)	1.00 (0.34–1.00)	0.80 (0.26–0.97)	1.00 (0.34–1.00)	1.00 (0.34–1.00)	1.00 (0.34–1.00)
SAH	0.50 (0.10–0.91)	1.00 (0.21–1.00)	0.67 (0.13–0.95)	0.50 (0.10–0.91)	1.00 (0.21–1.00)	0.67 (0.13–0.95)
Infarct demarcation	0.59 (0.36–0.78)	0.91 (0.62–0.98)	0.71 (0.46–0.87)	1.00 (0.74–1.00)	1.00 (0.74–1.00)	1.00 (0.74–1.00)
Vascular occlusion	1.00 (0.85–1.00)	1.00 (0.85–1.00)	1.00 (0.85–1.00)	1.00 (0.85–1.00)	1.00 (0.85–1.00)	1.00 (0.85–1.00)
Vascular stenosis	0.84 (0.67–0.93)	1.00 (0.87–1.00)	0.91 (0.76–0.96)	0.96 (0.81–0.99)	0.96 (0.81–0.99)	0.96 (0.81–0.99)
Aneurysm	0.67 (0.30–0.90)	1.00 (0.51–1.00)	0.80 (0.38–0.95)	0.80 (0.38–0.96)	1.00 (0.51–1.00)	0.89 (0.43–0.98)
Dissection	0.50 (0.10–0.91)	1.00 (0.21–1.00)	0.67 (0.13–0.95)	0.50 (0.10–0.91)	1.00 (0.21–1.00)	0.67 (0.13–0.95)
Ischemic perfusion deficit	0.96 (0.81–0.99)	0.96 (0.81–0.99)	0.96 (0.81–0.99)	1.00 (0.86–1.00)	0.96 (0.81–0.99)	0.98 (0.83–1.00)
Total	0.83 (0.75–0.89)	0.98 (0.93–0.99)	0.90 (0.83–0.94)	0.95 (0.89–0.98)	0.98 (0.93–0.99)	0.96 (0.91–0.99)

1.4% (14/1,000) of data points were excluded, as the report text indicated diagnostic uncertainty without a clear positive or negative tendency (expressions such as “possible”, “DDx”). Metrics for GPT-4o were calculated based on the mode across three repetitions

**Table 4:** Exemplary data extraction cases influenced by the annotation guideline (GPT-4o, dataset B, temperature = 1)

Parameter	Report excerpt	Relevant guideline instruction	Rating: GPT-4o – guideline	Rating: GPT-4o + guideline	Correct rating
SDH	No evidence of hyperdensities suggestive of bleeding. S/p left frontal trepanation for SDH.	Previous findings that are no longer present, are not considered.	Yes	No	No
Stenosis	Calcified plaques at the carotid bulb bilaterally.	A finding is only deemed present if it is explicitly mentioned. “Calcifications” or “plaques” are not equivalent to stenosis.	Yes	No	No
Stenosis	Caliber irregularities of the left vertebral artery with calcified plaque in the V4 segment. Along the right vertebral artery, multiple calcified plaques are observed.	“Calcifications” or “plaques” are not equivalent to stenosis. Vascular irregularities are not considered stenosis.	Yes	No	No
Infarct demarcation	Old partial infarction of the right MCA territory. No early signs of ischemia, no evidence of intracranial hemorrhage.	“Infarct demarcation” encompasses only acute changes. Chronic infarcts or post-ischemic changes shall not be counted.	Yes	No	No
Infarct demarcation	Hypodense parenchymal defect area in the right occipital lobe.	A finding is only deemed present if it is explicitly mentioned. Hypodensity in the parenchyma is not equivalent to infarct demarcation.	Yes	No	No
Aneurysm	Bilateral ICA pseudoaneurysms.	“Aneurysm” refers exclusively to true intracranial aneurysms. Aortic aneurysms or pseudoaneurysms shall not be classified as aneurysms.	Yes	No	No
Ischemic perfusion deficit	Slight Tmax delay in the left high frontal and high parietal regions with partially increased CBF, DDx postictal.	Only perfusion deficits attributed to ischemia in the findings text shall be counted. Perfusion deficits due to artifacts or other causes shall not be counted.	Yes	No	No

Report excerpts were translated from German to English

### Processing time and test-retest reliability

Mean processing times for GPT-4o (accessed via API) were 465.1 s per 100 reports, as compared to 1,441.4 s per 100 reports for Llama-3.3-70B (operated on a local GPU). The mean time for manual annotations (measured in dataset B) was considerably longer than both models (9,302.0 s per 100 reports). GPT-4o with a temperature setting of 1 featured a very high test-retest reliability, with identical ratings across three repetitions in 97.6% of data points.

### Diagnostic certainty assessment

GPT-4o accurately classified the diagnostic certainty level in 90.0% (900/1,000) of data points in dataset B. Yet, its performance was considerably lower in uncertain findings (categories ‘unlikely’, ‘possible’, ‘likely’), with only 35.0% (7/20) correct data points. In contrast, it performed markedly higher for certain findings (categories ‘definitely absent’, ‘definitely present’), achieving 91.1% accuracy (893/980).

## Discussion

In this study, we evaluated the performance of GPT-4o and Llama-3.3-70B in extracting data parameters from stroke CT reports with and without comprehensive annotation guidelines.

In summary, we demonstrate the promising performance of GPT-4o and Llama-3.3-70B in extracting findings from stroke CT reports. Our work extends previous studies illustrating the utility of LLMs in extracting data from mechanical thrombectomy reports [22, 25], brain MRI reports [24], and more. Although GPT-4o invariably outperformed Llama-3.3-70B under identical conditions, Llama-3.3-70B showed great potential, with overall precision and recall scores of up to 0.86 and 0.99, each. This is in accordance with several recent studies highlighting that open-source models are rapidly catching up with proprietary models [34–36]. Notably, numerous advantages of open-source models for clinical use have been pointed out, including enhanced data privacy, greater control over updates and customization, transparency, and stronger community collaboration [25, 37, 38]. Hence, it is reasonable to expect continued interest in and support for open-source models among the medical community, even though their local implementation demands great technical expertise and an advanced hardware infrastructure.

To explore the role of LLM temperature, a hyperparameter influencing output randomness and creativity, we performed GPT-4o queries with two different settings (0 and 1). but observed only negligible differences in data extraction metrics, consistent with prior research reporting stable data extraction performance in a temperature range from 0 to 1.5 [39]. Generally, higher temperature values increase output variability and creativity. In contrast, lower temperature yields more deterministic and consistent results, as well as fewer hallucinations (*i.e*., factually incorrect information), which might be preferable in medical applications where reliability and reproducibility are critical [40].

Crucially, this study emphasizes the role of a comprehensive annotation guideline on LLM data extraction performance. In both models and both datasets evaluated, the inclusion of a detailed annotation guideline led to an increase in precision, while retaining very high recall rates. The annotation guideline, which was equally adopted by the human annotators defining the reference standard, included definitions of the individual data variables, along with instructions for specific edge cases. A granular analysis on the variable level reveals that the improvement in data extraction metrics was primarily driven by a more precise and narrower definition of several key parameters, including ‘infarct demarcation’ and ‘vascular stenosis’.

The guideline additionally provided directions on handling diagnostic uncertainty of findings, an inherent limitation of diagnostic interpretations. A wide variability in phrases conveying certainty levels in radiology reports has been reported previously [41, 42]. While the uncertainty of findings cannot be eliminated, the annotation process requires categorization into a binary variable. To resolve this issue, our guideline specified that uncertain findings with a clear positive or negative tendency be classified as “Yes” or “No”, respectively, though equivocal findings (*e.g.*, “possible”, “DDx”) were manually excluded for the purpose of the analysis.

In a complementary experiment, we observed that GPT-4o displayed high accuracy in classifying findings on a 5-point certainty scale if findings were definitive (‘definitely present’, ‘definitely absent’) but struggled to correctly assign uncertain findings (‘likely’, ‘possible’, and ‘unlikely’), suggesting a potential weakness.

It is worth noting that the annotation guideline in this study was defined based on a meticulous review of cases with inter-annotator disagreement in one of the two datasets. This approach uncovered numerous edge cases and rating ambiguities that had not been anticipated in advance. When applying LLM-based data extraction in real-world use cases, annotation guidelines should be carefully designed to reflect the intended downstream use of the extracted data. For example, a more restrictive definition of variables leading to higher precision might be sensible if the accurate identification of certainly positive cases is decisive (*e.g.*, in a retrospective study with strict inclusion criteria), whereas higher sensitivity (recall) should be prioritized if the primary goal is not to miss any true positives (*e.g*., identifying critical incidents).

Despite the fast-paced advancement of LLM capabilities, data extraction from unstructured radiology reports is constrained by their lack of standardization of content and terminology. Classifications such as ASPECTS [43], which are frequently assessed in study settings, cannot be meaningfully analyzed if not routinely reported. Extracting the location of a finding is complicated by its variable description (*e.g*., in terms of adjacent structures, vascular territories, or brain lobes). Furthermore, findings that are not explicitly stated introduce another layer of ambiguity. In our study, findings were presumed absent unless explicitly mentioned, although in some cases, lacking mentions might be indicative of findings missed by the radiologist. The impact of this ambiguity on clinical communication was exemplified in a survey study, where half of the referring clinicians believed the radiologist might not have evaluated a particular finding if not explicitly documented [44].

The increased availability of LLMs for reliable and accurate data extraction from radiology reports has broad implications for the healthcare community. In contrast to the previously widespread practice of manual text annotation, LLMs offer a far more efficient and scalable approach to converting unstructured narrative reports into structured, machine-readable data. This capability facilitates the seamless integration of radiology findings into population health databases [45], supports the training of machine learning algorithms for the detection and classification of radiological abnormalities [46], and enables robust analytics for quality assurance and outcome monitoring [47].

Importantly, implementing a patient privacy-preserving workflow for LLM-powered data extraction from radiology reports would require either a secure cloud-based infrastructure, or on-premise hardware capable of running open-weight LLMs locally. While the study demonstrates the feasibility of using LLMs for automated data extraction, future work should address how this capability can be incorporated into existing radiology workflows. Potential integration pathways include embedding LLM services within radiology information systems (RIS), picture archiving and communication systems (PACS), or electronic health records (EHRs). However, clinical deployment would also require careful consideration of regulatory and ethical frameworks, including compliance with data protection regulations (*e.g*., General Data Protection Regulation—GDPR, Health Insurance Portability and Accountability Act—HIPAA) to mitigate bias and ensure patient safety.

Several limitations of this study need to be acknowledged. First, the single-center nature of this study necessitates further validation to confirm the generalizability of our findings across multiple institutions. Second, due to the relatively small sample size of the consecutive cohort and the low occurrence of some findings in the dataset, the variable-level analysis of data extraction metrics was underpowered. This may also explain why the improvement in precision in GPT-4o (temperature = 0) did not reach statistical significance, although the difference was notable (from 0.86 to 0.93). Third, this study utilized only German reports, and the influence of language on LLM performance has not been explicitly assessed. While language imbalances in the training data of the utilized LLMs are likely, only minor differences in model performance have been observed between high-resource languages such as English and German. For instance, GPT-4 demonstrated an accuracy of 85.5% on the Massive Multitask Language Understanding—MMLU benchmark in English, compared to 83.7% in German [48]. Therefore, it is unlikely that a similar dataset in English would lead to substantially different results. Finally, the performance of guideline-enhanced LLMs in dataset A needs to be interpreted with caution, given that the annotation guideline was derived from ambiguous cases within the same dataset.

In conclusion, our results demonstrate the potential of GPT-4o and Llama-3-70B in extracting key image findings from stroke CT reports, with GPT-4o steadily exceeding the performance of Llama-3-70B. We further provide evidence that well-defined annotation guidelines can enhance LLM data extraction accuracy.

## Supplementary information

**Supplemental material** Supplement 1: Parameters excluded from dataset A (translated from German to English). Supplement 2: Parameters excluded from Dataset B (translated from German to English). Supplement 3: Data extraction performance of GPT-4o (temperature = 1) with and without annotation guideline in dataset A (*n* = 200). Metrics for GPT-4o were calculated based on the mode across three repetitions. Supplement 4: Data extraction performance of GPT-4o (temperature = 1) with and without annotation guideline in dataset A (*n* = 200). Metrics for GPT-4o were calculated based on the mode across three repetitions. Supplement 5: Data extraction performance of Llama-3.3-70B (temperature = 0) with and without annotation guideline in dataset A (*n* = 200). Supplement 6: Data extraction performance of Llama-3.3-70B (temperature = 0) with and without annotation guideline in dataset A (*n* = 200). Supplement 7: Data extraction performance of Llama-3.3-70B (temperature = 0) with and without annotation guideline in dataset B (*n* = 100). Supplement 8: Data extraction performance of Llama-3.3-70B (temperature = 0) with and without annotation guideline in dataset B (*n* = 100). Supplement 9: Data extraction performance of GPT-4o (temperature = 0) with and without annotation guideline in dataset A (*n* = 100). Supplement 10: Data extraction performance of GPT-4o (temperature = 0) with and without annotation guideline in dataset A (*n* = 100). Supplement 11: Data extraction performance of GPT-4o (temperature = 0) with and without annotation guideline in dataset B (*n* = 100). Supplement 12: Data extraction performance of GPT-4o (temperature = 0) with and without annotation guideline in dataset B (*n* = 100). Supplement 13: data extraction performance of GPT-4o (temperature = 1) with and without annotation guideline in dataset B (*n* = 100).

## Data Availability

The code for running the LLM queries is provided at: https://github.com/shk03/stroke_llm_data_extraction.

## Abbreviations

ASPECTS: Alberta stroke program early CT score
DDx: Differential diagnosis
GPT-4o: Generative pre-trained transformer 4 omni
JSON: JavaScript object notation
Llama: Large language model meta AI
LLM: Large language model
