# Supplementary material for: Data extraction from free-text stroke CT reports using GPT-4o and Llama-3.3-70B: the impact of annotation guidelines
Source: Eur Radiol Exp. 2025 Jun 19;9:61. doi: 10.1186/s41747-025-00600-2 (PMC12179022; doi:10.1186/s41747-025-00600-2)
Supplement: Supplementary file 1 — Supplemental material Supplement 1: Parameters excluded from dataset A (translated from German to English). Supplement 2: Parameters excluded from Dataset B (translated from German to English). Supplement 3: Data extraction performance of GPT-4o (temperature = 1) with and without annotation guideline in dataset A (n = 200). Metrics for GPT-4o were calculated based on the mode across three repetitions. Supplement 4: Data extraction performance of GPT-4o (temperature = 1) with and without annotation guideline in dataset A (n = 200). Metrics for GPT-4o were calculated based on the mode across three repetitions. Supplement 5: Data extraction performance of Llama-3.3-70B (temperature = 0) with and without annotation guideline in dataset A (n = 200). Supplement 6: Data extraction performance of Llama-3.3-70B (temperature = 0) with and without annotation guideline in dataset A (n = 200). Supplement 7: Data extraction performance of Llama-3.3-70B (temperature = 0) with and without annotation guideline in dataset B (n = 100). Supplement 8: Data extraction performance of Llama-3.3-70B (temperature = 0) with and without annotation guideline in dataset B (n = 100). Supplement 9: Data extraction performance of GPT-4o (temperature = 0) with and without annotation guideline in dataset A (n = 100). Supplement 10: Data extraction performance of GPT-4o (temperature = 0) with and without annotation guideline in dataset A (n = 100). Supplement 11: Data extraction performance of GPT-4o (temperature = 0) with and without annotation guideline in dataset B (n = 100). Supplement 12: Data extraction performance of GPT-4o (temperature = 0) with and without annotation guideline in dataset B (n = 100). Supplement 13: data extraction performance of GPT-4o (temperature = 1) with and without annotation guideline in dataset B (n = 100). [file 41747_2025_600_MOESM1_ESM.pdf]

Data extraction from free-text stroke CT reports using GPT-4o and Llama-3.3-70B: the impact of annotation guidelines

ELECTRONIC SUPPLEMENTARY MATERIAL

Supplemental material

Case	Parameter	Report Excerpt
**A6**	Vascular occlusion	Possible occlusion of the left middle cerebral artery (MCA) in the distal M2 to proximal M3 segment of the inferior trunk, with associated perfusion delay.
**A16**	Infarct demarcation	Questionable demarcation is noted within the corresponding vascular territory; DDx chronic infarct.
**A18**	Intracerebral hemorrhage (ICH)	[…], faint hyperdensity is seen in the left frontal region; a small parenchymal hemorrhage cannot be definitively excluded.
**A22**	Dissection	Caliber irregularities observed in the proximal left internal carotid artery (ICA) without clear false lumen; overlapping artifacts present. DDx arterial dissection versus carotid web as potential embolic source.
**B14**	Vascular stenosis	The left vertebral artery appears slender compared to the contralateral side - possible stenosis. Irregularity of the basilar artery just distal to the vertebrobasilar confluence.
**B20**	Ischemic perfusion deficit	Perfusion: Tmax delay in the left cerebellum; DDx artifact-related delay.
**B23**	Vascular occlusion	Vessel discontinuation in the left P2 segment with re-opacification noted distal to the P3 segment onward. DDx short-segment occlusion DDx high-grade stenosis.
**B23**	Vascular stenosis	Vessel discontinuation in the left P2 segment with re-opacification noted distal to the P3 segment onward. DDx short-segment occlusion DDx high-grade stenosis.
**B25**	Vascular occlusion	Subtotal stenosis or occlusion of the left vertebral artery at the distal V4 segment.
**B28**	Infarct demarcation	Possible early infarct demarcation in the right PICA territory.
**B28**	Ischemic perfusion deficit	Possibly subtle perfusion delay in the right posterior cerebellar hemisphere.
**B35**	Ischemic perfusion deficit	CT perfusion is limited due to technical constraints.
**B38**	Ischemic perfusion deficit	CT perfusion: questionable slight Tmax elevation in the left basal cerebellum; otherwise unremarkable.
**B40**	Aneurysm	Vascular ectasia DDx aneurysm of the carotid T-junction.
**B40**	Ischemic perfusion deficit	Interpretation limited by due to technical constraints.
**C6**	Ischemic perfusion deficit	Diagnostic interpretability limited by motion artifacts; however, no major territorial perfusion deficit is seen. Possibly slight perfusion delay along the posterior watershed zone.
**C14**	Ischemic perfusion deficit	Perfusion: wedge-shaped delay in the left frontal region. DDx hypoperfusion DDx artifact.
**C24**	Infarct demarcation	Possible small subacute ischemic lesion in the left precentral gyrus.
**C28**	Ischemic perfusion deficit	CTP not available.
**C34**	Infarct demarcation	Questionable acute ischemic lesion in the right thalamus and left internal capsule.

**D1**	Intracerebral hemorrhage (ICH)	Acute blood accumulation is noted in the right parietal lobe, DDx sulcal subarachnoid hemorrhage, DDx parenchymal hemorrhage.
**D1**	Subarachnoid hemorrhage (SAH)	Acute blood accumulation is noted in the right parietal lobe, DDx sulcal subarachnoid hemorrhage, DDx parenchymal hemorrhage.
**D5**	Aneurysm	Approximately 1 cm, well-defined, peripherally calcified lesion in the right temporal pole.<br>DDx meningioma DDx fully thrombosed MCA aneurysm.
**D6**	Ischemic perfusion deficit	Questionable localized Tmax delay in the anterior left MCA territory; DDx artifact.
**D7**	Epidural hematoma (EDH)	Extra-axial, partially septated, dura-based lesion in the right frontoparietal region with mass effect on the central cortex […]. DDx encapsulated chronic epidural hematoma (trauma history?).
**D17**	Ischemic perfusion deficit	Perfusion imaging not interpretable due to motion artifacts.
**D18**	Intracerebral hemorrhage (ICH)	Small, rounded hyperdensity in the left temporal lobe; hemorrhage cannot be ruled out.
**D34**	Subarachnoid hemorrhage (SAH)	Focal hyperdensity seen in the left frontal region. DDx subarachnoid hemorrhage DDx parenchymal bleed.
**D40**	Ischemic perfusion deficit	Perfusion study is non-diagnostic due to technical limitations.
**E5**	Vascular stenosis	Small-caliber branch originating from the left A2 segment with short-segment caliber irregularity in its inferior course. DDx intracranial stenosis.
**E14**	Intracerebral hemorrhage (ICH)	Resorbed infarct scar in the right parietal region DDx cortical calcification or DDx small localized hemorrhage.
**E14**	Ischemic perfusion deficit	Questionable patchy perfusion delay in the right periventricular white matter, superior to the basal ganglia.
**E15**	Ischemic perfusion deficit	Interpretability limited by extensive motion artifacts.
**E24**	Ischemic perfusion deficit	In perfusion imaging, questionable slight Tmax delay along the right watershed zone.

Supplement 1: Parameters excluded from Dataset A (translated from German to English).

Case	Parameter	Report Excerpt
**10**	Subdural hematoma (SDH)	Narrow subdural fluid collection is noted over the right hemisphere without mass effect; DDx hygroma DDx chronic subdural hematoma.
**11**	Vascular occlusion	Stenosis or possible discontinuation of a very small, distal M2 branch over the right insula.
**17**	Vascular occlusion	The right vertebral artery shows loss of contrasting at the intracranial entry; DDx occlusion.
**23**	Epidural hematoma (EDH)	Bone flap in normal postoperative alignment; small subcalvarial blood collection is present, along with a narrow extracranial galea hematoma and postoperative air inclusions.
**23**	Subdural hematoma (SDH)	Bone flap in normal postoperative alignment; small subcalvarial blood collection is present, along with a narrow extracranial galea hematoma and postoperative air inclusions.
**23**	Subarachnoid hemorrhage (SAH)	Bone flap in normal postoperative alignment; small subcalvarial blood collection is present, along with a narrow extracranial galea hematoma and postoperative air inclusions.
**31**	Intracerebral hemorrhage (ICH)	Small acute hemorrhage is seen lateral to the left frontal horn of the lateral ventricle; DDx hemorrhagic lesion DDx traumatic shear injury.
**43**	Dissection	Irregularity of the left vertebral artery at the level of C3, with additional caliber variations in the V4 segment; DDx dissection DDx venous overlap artifact.
**50**	Vascular occlusion	Short-segment lack of contrast opacification in the P2 segment; DDx intraluminal thrombus.
**64**	Aneurysm	Questionable tiny basilar tip aneurysm DDx infundibulum.
**81**	Ischemic perfusion deficit	CTP not available.
**82**	Vascular occlusion	Questionable contrast discontinuation of a right frontal cortical MCA branch (M4 or more distal).
**83**	Ischemic perfusion deficit	CTP not available.
**87**	Dissection	Contrast discontinuation of the right internal carotid artery from the carotid bifurcation up to the intracranial entry; DDx dissection DDx thrombus.

Supplement 2: Parameters excluded from Dataset B (translated from German to English).

Variable	Dataset A: GPT-4o – Guideline (temp = 1)			Dataset A: GPT-4o + Guideline (temp = 1)		
	Precision	Recall	F1-Score	Precision	Recall	F1-Score
Intracerebral hemorrhage (ICH)	0.86 (0.65 to 0.95)	1.00 (0.82 to 1.00)	0.92 (0.73 to 0.97)	0.90 (0.70 to 0.97)	1.00 (0.82 to 1.00)	0.95 (0.76 to 0.99)
Epidural hematoma (EDH)	0.50 (0.10 to 0.91)	1.00 (0.21 to 1.00)	0.67 (0.13 to 0.95)	0.50 (0.10 to 0.91)	1.00 (0.21 to 1.00)	0.67 (0.13 to 0.95)
Subdural hematoma (SDH)	1.00 (0.81 to 1.00)	1.00 (0.81 to 1.00)	1.00 (0.81 to 1.00)	1.00 (0.81 to 1.00)	1.00 (0.81 to 1.00)	1.00 (0.81 to 1.00)
Subarachnoid hemorrhage (SAH)	0.94 (0.72 to 0.99)	0.94 (0.72 to 0.99)	0.94 (0.72 to 0.99)	0.94 (0.72 to 0.99)	0.94 (0.72 to 0.99)	0.94 (0.72 to 0.99)
Infarct demarcation	0.55 (0.43 to 0.66)	1.00 (0.90 to 1.00)	0.71 (0.58 to 0.79)	0.86 (0.72 to 0.93)	1.00 (0.90 to 1.00)	0.92 (0.80 to 0.97)
Vascular occlusion	0.99 (0.94 to 1.00)	0.99 (0.94 to 1.00)	0.99 (0.94 to 1.00)	0.99 (0.94 to 1.00)	0.98 (0.92 to 0.99)	0.98 (0.93 to 1.00)
Vascular stenosis	0.90 (0.81 to 0.94)	1.00 (0.95 to 1.00)	0.94 (0.88 to 0.97)	0.96 (0.90 to 0.99)	1.00 (0.95 to 1.00)	0.98 (0.92 to 0.99)
Aneurysm	0.90 (0.60 to 0.98)	1.00 (0.70 to 1.00)	0.95 (0.64 to 0.99)	1.00 (0.68 to 1.00)	0.89 (0.57 to 0.98)	0.94 (0.62 to 0.99)
Dissection	0.75 (0.30 to 0.95)	1.00 (0.44 to 1.00)	0.86 (0.36 to 0.98)	0.75 (0.30 to 0.95)	1.00 (0.44 to 1.00)	0.86 (0.36 to 0.98)
Ischemic perfusion deficit	0.95 (0.89 to 0.98)	0.98 (0.92 to 0.99)	0.96 (0.90 to 0.99)	0.94 (0.88 to 0.98)	0.99 (0.94 to 1.00)	0.97 (0.90 to 0.99)
Total	0.87 (0.83 to 0.90)	0.99 (0.97 to 0.99)	0.93 (0.90 to 0.94)	0.95 (0.92 to 0.96)	0.99 (0.97 to 0.99)	0.97 (0.94 to 0.98)

Supplement 3: Data extraction performance of GPT-4o (temperature = 1) with and without annotation guideline in dataset A (n = 200). Metrics for GPT-4o were calculated based on the mode across three repetitions.

Variable	Dataset A: GPT-4o – Guideline (temp = 1)		Dataset A: GPT-4o + Guideline (temp = 1)	
	NPV	Specificity	NPV	Specificity
Intracerebral hemorrhage (ICH)	1.00 (0.98 to 1.00)	0.98 (0.95 to 0.99)	1.00 (0.98 to 1.00)	0.99 (0.96 to 1.00)
Epidural hematoma (EDH)	1.00 (0.98 to 1.00)	0.99 (0.97 to 1.00)	1.00 (0.98 to 1.00)	0.99 (0.97 to 1.00)
Subdural hematoma (SDH)	1.00 (0.98 to 1.00)	1.00 (0.98 to 1.00)	1.00 (0.98 to 1.00)	1.00 (0.98 to 1.00)
Subarachnoid hemorrhage (SAH)	0.99 (0.97 to 1.00)	0.99 (0.97 to 1.00)	0.99 (0.97 to 1.00)	0.99 (0.97 to 1.00)
Infarct demarcation	1.00 (0.97 to 1.00)	0.81 (0.74 to 0.87)	1.00 (0.98 to 1.00)	0.96 (0.92 to 0.98)
Vascular occlusion	0.99 (0.95 to 1.00)	0.99 (0.95 to 1.00)	0.98 (0.94 to 1.00)	0.99 (0.95 to 1.00)
Vascular stenosis	1.00 (0.97 to 1.00)	0.93 (0.86 to 0.96)	1.00 (0.97 to 1.00)	0.97 (0.93 to 0.99)
Aneurysm	1.00 (0.98 to 1.00)	0.99 (0.97 to 1.00)	0.99 (0.97 to 1.00)	1.00 (0.98 to 1.00)
Dissection	1.00 (0.98 to 1.00)	0.99 (0.97 to 1.00)	1.00 (0.98 to 1.00)	0.99 (0.97 to 1.00)
Ischemic perfusion deficit	0.98 (0.93 to 0.99)	0.96 (0.90 to 0.98)	0.99 (0.94 to 1.00)	0.95 (0.89 to 0.98)
Total	1.00 (0.99 to 1.00)	0.97 (0.96 to 0.98)	1.00 (0.99 to 1.00)	0.99 (0.98 to 0.99)

Supplement 4: Data extraction performance of GPT-4o (temperature = 1) with and without annotation guideline in dataset A (n = 200). Metrics for GPT-4o were calculated based on the mode across three repetitions.

Variable	Dataset A: Llama-3.3-70B – Guideline (temp = 0)			Dataset A: Llama-3.3-70B + Guideline (temp = 0)		
	Precision	Recall	F1-Score	Precision	Recall	F1-Score
Intracerebral hemorrhage (ICH)	0.78 (0.58 to 0.90)	1.00 (0.82 to 1.00)	0.88 (0.68 to 0.95)	0.75 (0.55 to 0.88)	1.00 (0.82 to 1.00)	0.86 (0.66 to 0.94)
Epidural hematoma (EDH)	0.50 (0.10 to 0.91)	1.00 (0.21 to 1.00)	0.67 (0.13 to 0.95)	0.33 (0.06 to 0.79)	1.00 (0.21 to 1.00)	0.50 (0.09 to 0.88)
Subdural hematoma (SDH)	1.00 (0.81 to 1.00)	1.00 (0.81 to 1.00)	1.00 (0.81 to 1.00)	1.00 (0.81 to 1.00)	1.00 (0.81 to 1.00)	1.00 (0.81 to 1.00)
Subarachnoid hemorrhage (SAH)	0.94 (0.72 to 0.99)	0.94 (0.72 to 0.99)	0.94 (0.72 to 0.99)	0.94 (0.72 to 0.99)	0.94 (0.72 to 0.99)	0.94 (0.72 to 0.99)
Infarct demarcation	0.53 (0.41 to 0.64)	1.00 (0.90 to 1.00)	0.69 (0.57 to 0.78)	0.60 (0.47 to 0.71)	1.00 (0.90 to 1.00)	0.75 (0.62 to 0.83)
Vascular occlusion	0.86 (0.78 to 0.91)	1.00 (0.96 to 1.00)	0.92 (0.86 to 0.95)	0.98 (0.92 to 0.99)	0.98 (0.92 to 0.99)	0.98 (0.92 to 0.99)
Vascular stenosis	0.67 (0.58 to 0.75)	1.00 (0.95 to 1.00)	0.80 (0.72 to 0.86)	0.86 (0.77 to 0.91)	1.00 (0.95 to 1.00)	0.92 (0.85 to 0.95)
Aneurysm	0.90 (0.60 to 0.98)	1.00 (0.70 to 1.00)	0.95 (0.64 to 0.99)	1.00 (0.70 to 1.00)	1.00 (0.70 to 1.00)	1.00 (0.70 to 1.00)
Dissection	0.75 (0.30 to 0.95)	1.00 (0.44 to 1.00)	0.86 (0.36 to 0.98)	0.75 (0.30 to 0.95)	1.00 (0.44 to 1.00)	0.86 (0.36 to 0.98)
Ischemic perfusion deficit	0.93 (0.86 to 0.97)	0.99 (0.94 to 1.00)	0.96 (0.90 to 0.98)	0.91 (0.84 to 0.95)	0.99 (0.94 to 1.00)	0.95 (0.88 to 0.98)
Total	0.78 (0.74 to 0.81)	0.99 (0.98 to 1.00)	0.87 (0.84 to 0.90)	0.86 (0.82 to 0.89)	0.99 (0.97 to 0.99)	0.92 (0.89 to 0.94)

Supplement 5: Data extraction performance of Llama-3.3-70B (temperature = 0) with and without annotation guideline in dataset A (n = 200).

Variable	Dataset A: Llama-3.3-70B – Guideline (temp = 0)		Dataset A: Llama-3.3-70B + Guideline (temp = 0)	
	NPV	Specificity	NPV	Specificity
Intracerebral hemorrhage (ICH)	1.00<br>(0.98 to 1.00)	0.97<br>(0.94 to 0.99)	1.00<br>(0.98 to 1.00)	0.97<br>(0.93 to 0.98)
Epidural hematoma (EDH)	1.00<br>(0.98 to 1.00)	0.99<br>(0.97 to 1.00)	1.00<br>(0.98 to 1.00)	0.99<br>(0.96 to 1.00)
Subdural hematoma (SDH)	1.00<br>(0.98 to 1.00)	1.00<br>(0.98 to 1.00)	1.00<br>(0.98 to 1.00)	1.00<br>(0.98 to 1.00)
Subarachnoid hemorrhage (SAH)	0.99<br>(0.97 to 1.00)	0.99<br>(0.97 to 1.00)	0.99<br>(0.97 to 1.00)	0.99<br>(0.97 to 1.00)
Infarct demarcation	1.00<br>(0.97 to 1.00)	0.80<br>(0.73 to 0.85)	1.00<br>(0.97 to 1.00)	0.85<br>(0.79 to 0.90)
Vascular occlusion	1.00<br>(0.96 to 1.00)	0.88<br>(0.80 to 0.92)	0.98<br>(0.94 to 1.00)	0.98<br>(0.94 to 1.00)
Vascular stenosis	1.00<br>(0.96 to 1.00)	0.68<br>(0.60 to 0.76)	1.00<br>(0.97 to 1.00)	0.89<br>(0.82 to 0.94)
Aneurysm	1.00<br>(0.98 to 1.00)	0.99<br>(0.97 to 1.00)	1.00<br>(0.98 to 1.00)	1.00<br>(0.98 to 1.00)
Dissection	1.00<br>(0.98 to 1.00)	0.99<br>(0.97 to 1.00)	1.00<br>(0.98 to 1.00)	0.99<br>(0.97 to 1.00)
Ischemic perfusion deficit	0.99<br>(0.94 to 1.00)	0.94<br>(0.88 to 0.97)	0.99<br>(0.94 to 1.00)	0.92<br>(0.85 to 0.96)
Total	1.00<br>(1.00 to 1.00)	0.94<br>(0.93 to 0.95)	1.00<br>(0.99 to 1.00)	0.96<br>(0.95 to 0.97)

Supplement 6: Data extraction performance of Llama-3.3-70B (temperature = 0) with and without annotation guideline in dataset A (n = 200).

Variable	Dataset B: Llama-3.3-70B – Guideline (temp = 0)			Dataset B: Llama-3.3-70B + Guideline (temp = 0)		
	Precision	Recall	F1-Score	Precision	Recall	F1-Score
Intracerebral hemorrhage (ICH)	0.50 (0.21 to 0.79)	1.00 (0.51 to 1.00)	0.67 (0.30 to 0.88)	0.57 (0.25 to 0.84)	1.00 (0.51 to 1.00)	0.73 (0.34 to 0.91)
Epidural hematoma (EDH)	0.00 (0.00 to 0.00)	0.00 (0.00 to 0.00)	0.00 (0.00 to 0.00)	0.00 (0.00 to 0.79)	0.00 (0.00 to 0.00)	0.00 (0.00 to 0.00)
Subdural hematoma (SDH)	0.67 (0.21 to 0.94)	1.00 (0.34 to 1.00)	0.80 (0.26 to 0.97)	1.00 (0.34 to 1.00)	1.00 (0.34 to 1.00)	1.00 (0.34 to 1.00)
Subarachnoid hemorrhage (SAH)	0.50 (0.10 to 0.91)	1.00 (0.21 to 1.00)	0.67 (0.13 to 0.95)	0.50 (0.10 to 0.91)	1.00 (0.21 to 1.00)	0.67 (0.13 to 0.95)
Infarct demarcation	0.50 (0.31 to 0.69)	1.00 (0.74 to 1.00)	0.67 (0.43 to 0.82)	0.58 (0.36 to 0.77)	1.00 (0.74 to 1.00)	0.73 (0.49 to 0.87)
Vascular occlusion	0.84 (0.65 to 0.94)	1.00 (0.84 to 1.00)	0.91 (0.74 to 0.97)	0.95 (0.78 to 0.99)	1.00 (0.84 to 1.00)	0.98 (0.81 to 1.00)
Vascular stenosis	0.53 (0.39 to 0.66)	1.00 (0.87 to 1.00)	0.69 (0.54 to 0.80)	0.67 (0.51 to 0.79)	1.00 (0.87 to 1.00)	0.80 (0.64 to 0.89)
Aneurysm	0.67 (0.30 to 0.90)	1.00 (0.51 to 1.00)	0.80 (0.38 to 0.95)	0.80 (0.38 to 0.96)	1.00 (0.51 to 1.00)	0.89 (0.43 to 0.98)
Dissection	0.50 (0.10 to 0.91)	1.00 (0.21 to 1.00)	0.67 (0.13 to 0.95)	0.50 (0.10 to 0.91)	1.00 (0.21 to 1.00)	0.67 (0.13 to 0.95)
Ischemic perfusion deficit	0.86 (0.69 to 0.94)	0.96 (0.81 to 0.99)	0.91 (0.74 to 0.97)	0.85 (0.68 to 0.94)	0.92 (0.75 to 0.98)	0.89 (0.71 to 0.96)
Total	0.65 (0.57 to 0.72)	0.99 (0.94 to 1.00)	0.78 (0.71 to 0.84)	0.74 (0.66 to 0.81)	0.98 (0.93 to 0.99)	0.84 (0.77 to 0.89)

Supplement 7: Data extraction performance of Llama-3.3-70B (temperature = 0) with and without annotation guideline in dataset B (n = 100).

Variable	Dataset B: Llama-3.3-70B – Guideline (temp = 0)		Dataset B: Llama-3.3-70B + Guideline (temp = 0)	
	NPV	Specificity	NPV	Specificity
Intracerebral hemorrhage (ICH)	1.00 (0.96 to 1.00)	0.96 (0.90 to 0.98)	1.00 (0.96 to 1.00)	0.97 (0.91 to 0.99)
Epidural hematoma (EDH)	1.00 (0.96 to 1.00)	1.00 (0.96 to 1.00)	1.00 (0.96 to 1.00)	0.99 (0.94 to 1.00)
Subdural hematoma (SDH)	1.00 (0.96 to 1.00)	0.99 (0.94 to 1.00)	1.00 (0.96 to 1.00)	1.00 (0.96 to 1.00)
Subarachnoid hemorrhage (SAH)	1.00 (0.96 to 1.00)	0.99 (0.94 to 1.00)	1.00 (0.96 to 1.00)	0.99 (0.94 to 1.00)
Infarct demarcation	1.00 (0.95 to 1.00)	0.88 (0.79 to 0.93)	1.00 (0.95 to 1.00)	0.91 (0.83 to 0.95)
Vascular occlusion	1.00 (0.95 to 1.00)	0.95 (0.87 to 0.98)	1.00 (0.95 to 1.00)	0.99 (0.93 to 1.00)
Vascular stenosis	1.00 (0.93 to 1.00)	0.69 (0.58 to 0.78)	1.00 (0.94 to 1.00)	0.82 (0.72 to 0.89)
Aneurysm	1.00 (0.96 to 1.00)	0.98 (0.93 to 0.99)	1.00 (0.96 to 1.00)	0.99 (0.94 to 1.00)
Dissection	1.00 (0.96 to 1.00)	0.99 (0.94 to 1.00)	1.00 (0.96 to 1.00)	0.99 (0.94 to 1.00)
Ischemic perfusion deficit	0.99 (0.92 to 1.00)	0.95 (0.87 to 0.98)	0.97 (0.90 to 0.99)	0.95 (0.87 to 0.98)
Total	1.00 (0.99 to 1.00)	0.94 (0.93 to 0.96)	1.00 (0.99 to 1.00)	0.96 (0.95 to 0.97)

Supplement 8: Data extraction performance of Llama-3.3-70B (temperature = 0) with and without annotation guideline in dataset B (n = 100).

Variable	Dataset A: GPT-4o – Guideline (temp = 0)			Dataset A: GPT-4o + Guideline (temp = 0)		
	Precision	Recall	F1-Score	Precision	Recall	F1-Score
Intracerebral hemorrhage (ICH)	0.86<br>(0.65 to 0.95)	1.00<br>(0.82 to 1.00)	0.92<br>(0.73 to 0.97)	0.90<br>(0.70 to 0.97)	1.00<br>(0.82 to 1.00)	0.95<br>(0.76 to 0.99)
Epidural hematoma (EDH)	0.50<br>(0.10 to 0.91)	1.00<br>(0.21 to 1.00)	0.67<br>(0.13 to 0.95)	0.50<br>(0.10 to 0.91)	1.00<br>(0.21 to 1.00)	0.67<br>(0.13 to 0.95)
Subdural hematoma (SDH)	1.00<br>(0.81 to 1.00)	1.00<br>(0.81 to 1.00)	1.00<br>(0.81 to 1.00)	1.00<br>(0.81 to 1.00)	1.00<br>(0.81 to 1.00)	1.00<br>(0.81 to 1.00)
Subarachnoid hemorrhage (SAH)	0.94<br>(0.72 to 0.99)	0.94<br>(0.72 to 0.99)	0.94<br>(0.72 to 0.99)	1.00<br>(0.80 to 1.00)	0.94<br>(0.72 to 0.99)	0.97<br>(0.75 to 0.99)
Infarct demarcation	0.52<br>(0.41 to 0.64)	1.00<br>(0.90 to 1.00)	0.69<br>(0.56 to 0.78)	0.84<br>(0.70 to 0.92)	1.00<br>(0.90 to 1.00)	0.91<br>(0.79 to 0.96)
Vascular occlusion	0.99<br>(0.94 to 1.00)	0.99<br>(0.94 to 1.00)	0.99<br>(0.94 to 1.00)	0.99<br>(0.94 to 1.00)	0.99<br>(0.94 to 1.00)	0.99<br>(0.94 to 1.00)
Vascular stenosis	0.89<br>(0.80 to 0.94)	1.00<br>(0.95 to 1.00)	0.94<br>(0.87 to 0.97)	0.97<br>(0.91 to 0.99)	0.96<br>(0.89 to 0.99)	0.97<br>(0.90 to 0.99)
Aneurysm	0.90<br>(0.60 to 0.98)	1.00<br>(0.70 to 1.00)	0.95<br>(0.64 to 0.99)	1.00<br>(0.70 to 1.00)	1.00<br>(0.70 to 1.00)	1.00<br>(0.70 to 1.00)
Dissection	0.75<br>(0.30 to 0.95)	1.00<br>(0.44 to 1.00)	0.86<br>(0.36 to 0.98)	0.75<br>(0.30 to 0.95)	1.00<br>(0.44 to 1.00)	0.86<br>(0.36 to 0.98)
Ischemic perfusion deficit	0.94<br>(0.87 to 0.98)	0.98<br>(0.92 to 0.99)	0.96<br>(0.90 to 0.98)	0.94<br>(0.88 to 0.98)	1.00<br>(0.96 to 1.00)	0.97<br>(0.92 to 0.99)
Total	0.86<br>(0.82 to 0.89)	0.99<br>(0.97 to 1.00)	0.92<br>(0.89 to 0.94)	0.95<br>(0.92 to 0.97)	0.99<br>(0.97 to 0.99)	0.97<br>(0.94 to 0.98)

Supplement 9: Data extraction performance of GPT-4o (temperature = 0) with and without annotation guideline in dataset A (n = 100).

Variable	Dataset A: GPT-4o – Guideline (temp = 0)		Dataset A: GPT-4o + Guideline (temp = 0)	
	NPV	Specificity	NPV	Specificity
Intracerebral hemorrhage (ICH)	1.00 (0.98 to 1.00)	0.98 (0.95 to 0.99)	1.00 (0.98 to 1.00)	0.99 (0.96 to 1.00)
Epidural hematoma (EDH)	1.00 (0.98 to 1.00)	0.99 (0.97 to 1.00)	1.00 (0.98 to 1.00)	0.99 (0.97 to 1.00)
Subdural hematoma (SDH)	1.00 (0.98 to 1.00)	1.00 (0.98 to 1.00)	1.00 (0.98 to 1.00)	1.00 (0.98 to 1.00)
Subarachnoid hemorrhage (SAH)	0.99 (0.97 to 1.00)	0.99 (0.97 to 1.00)	0.99 (0.97 to 1.00)	1.00 (0.98 to 1.00)
Infarct demarcation	1.00 (0.97 to 1.00)	0.79 (0.72 to 0.85)	1.00 (0.98 to 1.00)	0.96 (0.91 to 0.98)
Vascular occlusion	0.99 (0.95 to 1.00)	0.99 (0.95 to 1.00)	0.99 (0.95 to 1.00)	0.99 (0.95 to 1.00)
Vascular stenosis	1.00 (0.97 to 1.00)	0.92 (0.85 to 0.95)	0.98 (0.93 to 0.99)	0.98 (0.94 to 1.00)
Aneurysm	1.00 (0.98 to 1.00)	0.99 (0.97 to 1.00)	1.00 (0.98 to 1.00)	1.00 (0.98 to 1.00)
Dissection	1.00 (0.98 to 1.00)	0.99 (0.97 to 1.00)	1.00 (0.98 to 1.00)	0.99 (0.97 to 1.00)
Ischemic perfusion deficit	0.98 (0.93 to 0.99)	0.95 (0.89 to 0.98)	1.00 (0.96 to 1.00)	0.95 (0.89 to 0.98)
Total	1.00 (0.99 to 1.00)	0.97 (0.96 to 0.97)	1.00 (0.99 to 1.00)	0.99 (0.98 to 0.99)

Supplement 10: Data extraction performance of GPT-4o (temperature = 0) with and without annotation guideline in dataset A (n = 100).

Variable	Dataset B: GPT-4o – Guideline (temp = 0)			Dataset B: GPT-4o + Guideline (temp = 0)		
	Precision	Recall	F1-Score	Precision	Recall	F1-Score
Intracerebral hemorrhage (ICH)	1.00 (0.51 to 1.00)	1.00 (0.51 to 1.00)	1.00 (0.51 to 1.00)	1.00 (0.51 to 1.00)	1.00 (0.51 to 1.00)	1.00 (0.51 to 1.00)
Epidural hematoma (EDH)	0.00 (0.00 to 0.00)	0.00 (0.00 to 0.00)	0.00 (0.00 to 0.00)	0.00 (0.00 to 0.00)	0.00 (0.00 to 0.00)	0.00 (0.00 to 0.00)
Subdural hematoma (SDH)	0.67 (0.21 to 0.94)	1.00 (0.34 to 1.00)	0.80 (0.26 to 0.97)	1.00 (0.34 to 1.00)	1.00 (0.34 to 1.00)	1.00 (0.34 to 1.00)
Subarachnoid hemorrhage (SAH)	0.50 (0.10 to 0.91)	1.00 (0.21 to 1.00)	0.67 (0.13 to 0.95)	0.50 (0.10 to 0.91)	1.00 (0.21 to 1.00)	0.67 (0.13 to 0.95)
Infarct demarcation	0.65 (0.41 to 0.83)	1.00 (0.74 to 1.00)	0.79 (0.53 to 0.91)	0.85 (0.58 to 0.96)	1.00 (0.74 to 1.00)	0.92 (0.65 to 0.98)
Vascular occlusion	1.00 (0.85 to 1.00)	1.00 (0.85 to 1.00)	1.00 (0.85 to 1.00)	1.00 (0.85 to 1.00)	1.00 (0.85 to 1.00)	1.00 (0.85 to 1.00)
Vascular stenosis	0.84 (0.67 to 0.93)	1.00 (0.87 to 1.00)	0.91 (0.76 to 0.96)	0.96 (0.81 to 0.99)	0.96 (0.81 to 0.99)	0.96 (0.81 to 0.99)
Aneurysm	0.67 (0.30 to 0.90)	1.00 (0.51 to 1.00)	0.80 (0.38 to 0.95)	0.80 (0.38 to 0.96)	1.00 (0.51 to 1.00)	0.89 (0.43 to 0.98)
Dissection	0.50 (0.10 to 0.91)	1.00 (0.21 to 1.00)	0.67 (0.13 to 0.95)	0.50 (0.10 to 0.91)	1.00 (0.21 to 1.00)	0.67 (0.13 to 0.95)
Ischemic perfusion deficit	1.00 (0.86 to 1.00)	0.96 (0.81 to 0.99)	0.98 (0.83 to 1.00)	0.96 (0.81 to 0.99)	0.96 (0.81 to 0.99)	0.96 (0.81 to 0.99)
Total	0.86 (0.78 to 0.91)	0.99 (0.94 to 1.00)	0.92 (0.85 to 0.95)	0.93 (0.86 to 0.97)	0.98 (0.93 to 0.99)	0.95 (0.89 to 0.98)

Supplement 11: Data extraction performance of GPT-4o (temperature = 0) with and without annotation guideline in dataset B (n = 100).

Variable	Dataset B: GPT-4o – Guideline (temp = 0)		Dataset B: GPT-4o + Guideline (temp = 0)	
	NPV	Specificity	NPV	Specificity
Intracerebral hemorrhage (ICH)	1.00<br>(0.96 to 1.00)	1.00<br>(0.96 to 1.00)	1.00<br>(0.96 to 1.00)	1.00<br>(0.96 to 1.00)
Epidural hematoma (EDH)	1.00<br>(0.96 to 1.00)	1.00<br>(0.96 to 1.00)	1.00<br>(0.96 to 1.00)	1.00<br>(0.96 to 1.00)
Subdural hematoma (SDH)	1.00<br>(0.96 to 1.00)	0.99<br>(0.94 to 1.00)	1.00<br>(0.96 to 1.00)	1.00<br>(0.96 to 1.00)
Subarachnoid hemorrhage (SAH)	1.00<br>(0.96 to 1.00)	0.99<br>(0.94 to 1.00)	1.00<br>(0.96 to 1.00)	0.99<br>(0.94 to 1.00)
Infarct demarcation	1.00<br>(0.96 to 1.00)	0.93<br>(0.86 to 0.97)	1.00<br>(0.96 to 1.00)	0.98<br>(0.92 to 0.99)
Vascular occlusion	1.00<br>(0.95 to 1.00)	1.00<br>(0.95 to 1.00)	1.00<br>(0.95 to 1.00)	1.00<br>(0.95 to 1.00)
Vascular stenosis	1.00<br>(0.95 to 1.00)	0.93<br>(0.85 to 0.97)	0.99<br>(0.93 to 1.00)	0.99<br>(0.93 to 1.00)
Aneurysm	1.00<br>(0.96 to 1.00)	0.98<br>(0.93 to 0.99)	1.00<br>(0.96 to 1.00)	0.99<br>(0.94 to 1.00)
Dissection	1.00<br>(0.96 to 1.00)	0.99<br>(0.94 to 1.00)	1.00<br>(0.96 to 1.00)	0.99<br>(0.94 to 1.00)
Ischemic perfusion deficit	0.99<br>(0.93 to 1.00)	1.00<br>(0.95 to 1.00)	0.99<br>(0.93 to 1.00)	0.99<br>(0.93 to 1.00)
Total	1.00<br>(0.99 to 1.00)	0.98<br>(0.97 to 0.99)	1.00<br>(0.99 to 1.00)	0.99<br>(0.98 to 1.00)

Supplement 12: Data extraction performance of GPT-4o (temperature = 0) with and without annotation guideline in dataset B (n = 100).

Variable	Dataset B: GPT-4o – Guideline (temp = 1)		Dataset B: GPT-4o + Guideline (temp = 1)	
	NPV	Specificity	NPV	Specificity
Intracerebral hemorrhage (ICH)	1.00 (0.96 to 1.00)	0.99 (0.94 to 1.00)	1.00 (0.96 to 1.00)	0.99 (0.94 to 1.00)
Epidural hematoma (EDH)	1.00 (0.96 to 1.00)	1.00 (0.96 to 1.00)	1.00 (0.96 to 1.00)	1.00 (0.96 to 1.00)
Subdural hematoma (SDH)	1.00 (0.96 to 1.00)	0.99 (0.94 to 1.00)	1.00 (0.96 to 1.00)	1.00 (0.96 to 1.00)
Subarachnoid hemorrhage (SAH)	1.00 (0.96 to 1.00)	0.99 (0.94 to 1.00)	1.00 (0.96 to 1.00)	0.99 (0.94 to 1.00)
Infarct demarcation	0.99 (0.93 to 1.00)	0.92 (0.85 to 0.96)	1.00 (0.96 to 1.00)	1.00 (0.96 to 1.00)
Vascular occlusion	1.00 (0.95 to 1.00)	1.00 (0.95 to 1.00)	1.00 (0.95 to 1.00)	1.00 (0.95 to 1.00)
Vascular stenosis	1.00 (0.95 to 1.00)	0.93 (0.85 to 0.97)	0.99 (0.93 to 1.00)	0.99 (0.93 to 1.00)
Aneurysm	1.00 (0.96 to 1.00)	0.98 (0.93 to 0.99)	1.00 (0.96 to 1.00)	0.99 (0.94 to 1.00)
Dissection	1.00 (0.96 to 1.00)	0.99 (0.94 to 1.00)	1.00 (0.96 to 1.00)	0.99 (0.94 to 1.00)
Ischemic perfusion deficit	0.99 (0.93 to 1.00)	0.99 (0.93 to 1.00)	0.99 (0.93 to 1.00)	1.00 (0.95 to 1.00)
Total	1.00 (0.99 to 1.00)	0.98 (0.97 to 0.99)	1.00 (0.99 to 1.00)	0.99 (0.99 to 1.00)

Supplement 13: Data extraction performance of GPT-4o (temperature = 1) with and without annotation guideline in dataset B (n = 100).
